# Friction Stir Welding of Thick Plates of 4Y3Gd Mg Alloy: An Investigation of Microstructure and Mechanical Properties

**DOI:** 10.3390/ma14226924

**Published:** 2021-11-16

**Authors:** Khaja Moiduddin, Arshad Noor Siddiquee, Mustufa Haider Abidi, Syed Hammad Mian, Muneer Khan Mohammed

**Affiliations:** 1 Advanced Manufacturing Institute, King Saud University, Riyadh 11421, Saudi Arabia; mabidi@ksu.edu.sa (M.H.A.); smien@ksu.edu.sa (S.H.M.); muneerkm@ksu.edu.sa (M.K.M.); 2 Department of Mechanical Engineering, Faculty of Engineering and Technology, Jamia Millia Islamia, New Delhi 110025, India

**Keywords:** light-weight alloys, aerospace alloys, friction stir welding, non-ferrous metals

## Abstract

Applications of non-ferrous light metal alloys are especially popular in the field of aerospace. Hence it is important to investigate their properties in joining processes such as welding. Solid state joining process such as friction stir welding (FSW) is quite efficient for joining non-ferrous alloys, but with thick plates, challenges increase. In this study, Mg alloy plates of thickness 11.5 mm were successfully welded via single-pass FSW. Due to the dynamic recrystallization, grain size in the stir zone was reduced to 16 µm which is ≈15 times smaller than the parent material. The optimized rotational speed and traverse speed for optimum weld integrity were found to be 710 rpm and 100 mm/min, respectively. A sound weld with 98.96% joint efficiency, having an Ultimate Tensile Strength (UTS) of 161.8 MPa and elongation of 27.83%, was accomplished. Microhardness of the nugget was increased by 14.3%.

## 1. Introduction

The better forming methods and developments in the anti-corrosion treatment of Mg-alloys have significantly increased the acceptability of Mg-alloys for widespread industrial applications, including in the space/aerospace and automotive industries [1]. The Mg-alloys have already occupied more than 15% of vehicle weight in the past decade alone [2]. Although the fuel-saving, reduction in carbon footprint has remained the primary driving force behind the expanse of this light metal, which is 33% lighter than aluminum, the growth in the electric vehicle (EV) segments adds another dimension. Apart from the reduced energy consumption, it will enhance the mileage-per-battery-recharge and reduce the charging per unit km of travel. The Mg alloys are projected as one of the most promising materials for the transportation sector, including aerial road and rail-road transportations [3]. Typical applications in automobiles include clutch and transmission housing, valve cover, crankcase, engine cover, and cylinder head. Such products have a high thickness shell configuration. Other applications include steering wheels, steering brackets, brake brackets, seat frames, and alloy wheel reams. The wheel ream is typically homogenized and strengthened by friction stir processing (FSP) [4]. The electromagnetic shielding ability and noise damping capacity of Mg-alloys make them attractive candidates for defense applications too [5]. The space and aerospace applications include brackets and ribs, turbofans, an auxiliary casing of the turbo-engine, and a host of other heavy products [6,7]. Despite the potential for large-scale applications, the issues of drops in strength and poor ductility are some of the most critical barriers which limit the large-scale industrial application of such ultra-light alloys [8]. Addition of rare-earth (RE) elements (such as Zr, Gd, Y, Ba, and Sm) through casting route results in significant improvements in the corrosion and mechanical behavior of Mg-alloys. As the RE element additions in the Mg-alloys are newer, most welding is reported on conventional alloys, and welding of RE-based alloys has yet to pick up [9]. 

The fast expanse of Mg-alloys has opened new challenges for welding of thick sections as the welding of Mg-alloys with thickness greater than 10 mm is rarely reported, barring a very few made by electron beam welding process [10]. Conventional methods such as gas tungsten arc welding (GTAW) and high energy beam welding such as electron beam welding produce successful welds, but the joint efficiency is of the order of 70% and vacuum/high purity inert gas shielding is required to safeguard against high reactivity of Mg from the atmosphere [10]. Most literature reporting welding of Mg alloys with high thicknesses (8–10 mm thick) utilize high energy beam welding processes such as laser and electron beam welding [11]. These processes, apart from limitations of high capital cost, strict fit-up requirements, use of vacuum, etc., work with very intense heat. The low boiling point of Mg causes loss of alloying elements during welding at intense heat input. Furthermore, due to strict fit-ups, most literature reports the work on bead-on-plate welding. Further applications in the transportation sector will open up only after joining of thick Mg-alloy sections is matured. 

The issues of loss of alloying elements, needed for close fit-up, and problems due to strong basal texture can all be addressed satisfactorily via Friction Stir Welding (FSW) provided that the technology to weld thick section Mg-alloys is well evaluated. The solid-state friction stir welds have showed an efficiency of the order of 90% [12]. Lately, FSW of thick sections has also been explored by researchers [13,14,15,16]. For AA7075 aluminum alloy, Rao et al. [13] attained a joint efficiency of 70% and 53% for 10 mm and 16 mm plate thickness, respectively. Comparisons between FSW and underwater FSW for 19 mm thick AA2519 alloy have also been performed [14]. Further, the impact of tool design and process parameters for thick section FSW of aluminum alloys has also been addressed [15,16]. Due to increase in plate thickness, thermal disparity across the weld depth is found to influence mechanical and microstructural features in AA2219 alloys [17]. Owing to the problems associated with thick section FSW, such as tool pin failure and insufficient heat at the weld bottom, double side and bobbin tool FSW have also been scrutinized by researchers [18,19,20]. Employing asymmetric tool geometry on both sides of the plate’s surface in double side FSW, even for 3 mm thick flats, has proved to be beneficial to texture evolution [21]. However, any deviation from the simple one-sided single pass FSW (such as double-sided, and multiple pass welds) involves a significant increase in throughput time because of an increase in set-up time due to tool and workpiece cooling, clamping/de-clamping, enhanced set-up time, inter-pass correction of bead surface between subsequent passes, etc. 

It is evident from the literature review that considerable developments have been made in the FSW of thick section aluminum alloys. On the other hand, the FSW of thick Mg extruded flats poses much greater difficulty because of the brittle nature of Mg alloys. Yet, progress towards thick section FSW of magnesium alloys is also being made, as few studies have recently been reported in this regard [22]. Such progress is largely directed towards weight reduction in aircrafts and automobiles to improve fly-to-buy ratios and fuel economies. To address this need to foster welding of thick section Mg-alloys, the 11.5 mm thick Mg-alloy plates were butt-welded by friction stir welding. The effect of process parameters on the weld joint in a single pass FSW was investigated in this maiden work. The passes were planned so that higher weld quality with higher joint efficiency could be achieved in a single pass. This work is not only expected to demonstrate the desired weld strategy for thick Mg-alloy but to also provide a guiding framework for the welding of even greater thickness Mg-alloys for real applications in the strategic transportation, defense, and armor sectors.

## 2. Materials and Methods

**Figure 1:**
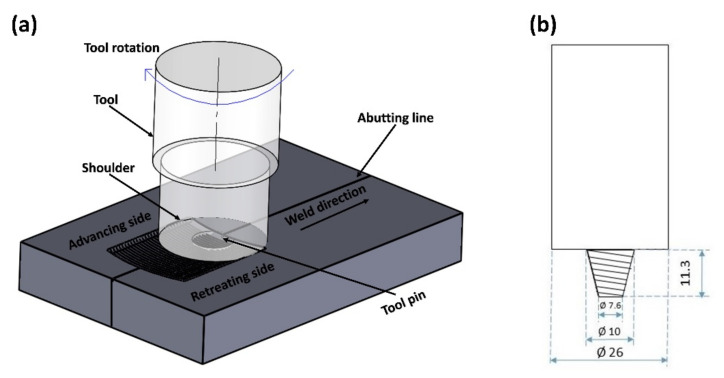
Schematic representation of (**a**) FSW Experimental Setup and (**b**) Tool design (all dimensions are in ‘mm’).

The WE-43 Mg alloy plates of thickness 11.5 mm were FS welded. Table 1 represents the chemical composition of the major elements provided by the supplier and mechanical properties of the base alloy. Four different experiments with a different set of process parameters, as per Taguchi’s L4 orthogonal array, were carried out to achieve the friction stir welds, as shown in Table 2. The FSW parameter window employed was based upon prior extensive trial experimentation to obtain welds with good surface finish and no craters. The parameters outside the presented processing window were found to yield very large surface craters and minimal joining during trial experimentation. To present the effect of process parameters in a justified manner, Taguchi’s L4 orthogonal array was extracted from the parameter window and investigated for microstructural and mechanical characterization. A High-Speed Steel (HSS) tool, having tapered cam tri-flute pin geometry with a left-hand thread of pitch 1 mm, was utilized for FSW. The shoulder diameter, pin tip diameter, pin root diameter, and pin length were set as 26 mm, 7.6 mm, 10 mm, and 11.2 mm, respectively. A tool tilt angle of 2⁰ was maintained. The experiments were carried out on a robust vertical milling machine adapted for performing FSW through an in-house designed and fabricated tool adaptor and work fixture. The FSW experimentation in the presented study was displacement-controlled. Figure 1 represents the schematic diagrams of FSW setup and tool design.

**Figure 2:**
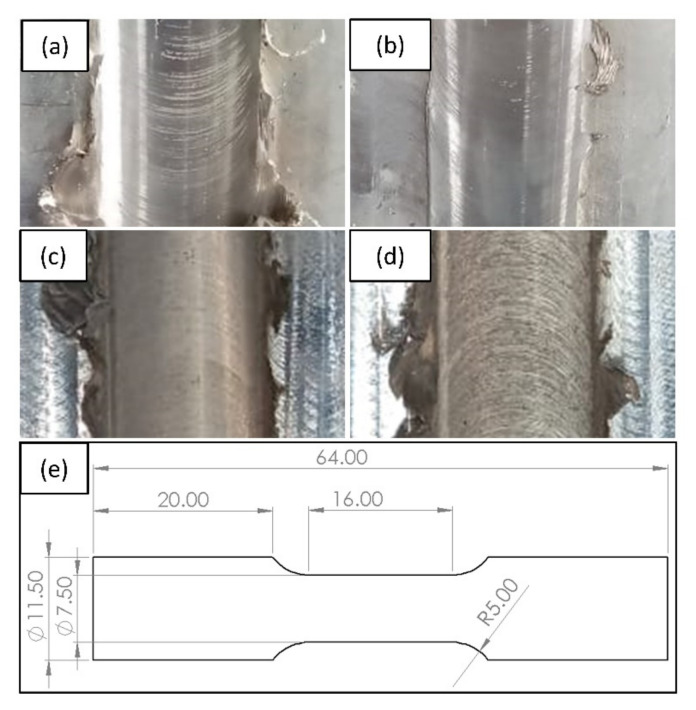
(**a**–**d**) Weldments corresponding to Exp. Nos. 1–4 (**e**) Schematic diagram of as machined tensile specimen (all dimensions are in ‘mm’).

To observe the surface topography and weld quality, samples for microstructure and tensile testing were drawn via wire electric discharge machining. The tensile specimens were machined according to the sub sized ASTM E8M standards. After standard metallographic procedures, samples were etched with a solution of 11 gm picric acid, 11 mL acetic acid, and 100 mL ethanol for 60 s. An Optical Microscope (OM; make: QS Meteorology) having the magnifications 100, 200, 400, 600, and 1000 × was employed for analysis of defects in weld zones (SZ, TMAZ, and HAZ) and evaluation of grain size distributions. The grain size was measured by line-intercept method using the Metallurgical Image Analysis Software (MIAS) ImageJ. Line intercepts were taken along 0, 45, 90, and 135° and averaged for the measurement of grain size. Further, micro-hardness results of the base metal and the welded specimen were obtained via a Mitutoyo Vickers Micro-Hardness Tester (Mitutoyo, Kawasaki, Japan). The micro-hardness measurements were taken at a distance of 4.5 mm from the top surface of welds. A Tensometer (Kudale Instruments Pvt Ltd, Pune, India) of capacity 20 kN was employed for performing the tensile examination of the base metal and the friction stir welds. The employed Tensometer included load and displacement sensors for measurement of force and strain. The real time force and strain values were read through a computer interfaced data acquisition system. The values of force were converted to stresses through the area of cross-section provided as input to the software system. Figure 2 shows the weldments corresponding to Exp. Nos. 1–4 and the dimensions of as-machined tensile specimens. 

## 3. Results and Discussion

### 3.1. Macrostructure

**Figure 3:**
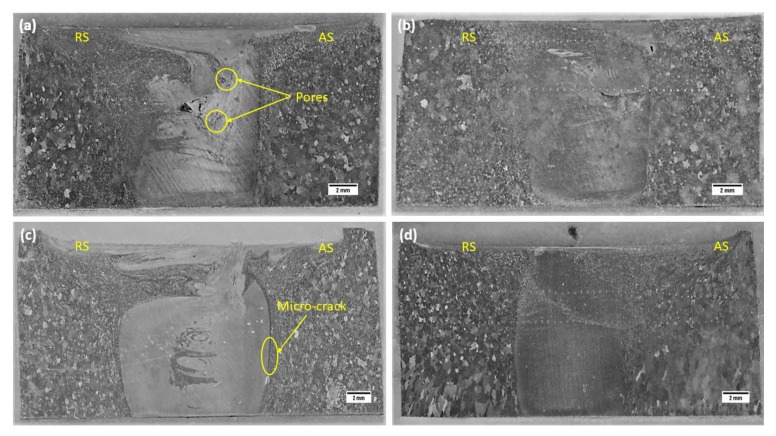
Macrostructures corresponding to (**a**) Exp. No. 1, (**b**) Exp. No. 2, (**c**) Exp. No. 3, and (**d**) Exp. No. 4.

The macrostructure of various FS welds, as shown in Figure 3, clearly demarcates the well-known characteristic zones of the FS welds, namely the Stir Zone (SZ)/nugget zone, Thermo-Mechanically Affected Zone (TMAZ), and the Heat Affected Zone (HAZ). Subject to varying strain and temperature during welding, each zone has specific characteristic properties and grain size. The nugget zone experiences severe plastic deformation (SPD) and high-temperature thermal cycles due to the direct stirring by the tool pin, resulting in dynamic recrystallization (DRX). The plastic deformation in TMAZ is the result of induced strain, strain rates, and lower temperature. HAZ experiences no plastic deformation, undergoes even colder thermal cycles, and generally has a larger grain size than the base alloy.

Macro-structures corresponding to Exp. No. 1 (Figure 3a) and Exp. No. 2 (Figure 3b) show the presence of tunnelling defects. In most of the studies reported earlier, the tunnels are usually formed on the advancing side (AS) in the bottom of the weld, mainly due to insufficient material flow and mixing. The prime cause for the same is insufficient heat input [23,24,25]. During FSW, the heat input is controlled by $\frac{\omega^{2}}{V}$ ratio (pseudo heat index) and its higher value indicates larger heat input [26]. In the present study, the tunnel defects/voids are found on the interface of the shoulder affected SZ and pin affected SZ. Due to higher weld thickness, it is difficult to maintain thermal and mechanical synergy across the weld depth, leading to defects at this location. The temperature gradient also becomes steeper along the weld depth, resulting in fluctuation of flow stress. For Exp. No. 1, heat input is maximum due to higher rotational speed and lower traverse speed. Therefore, the interface of hotter/softer shoulder affected SZ and colder/harder pin affected SZ becomes susceptible to poor material flow and largest tunnel defect is observed for Exp. No. 1. In addition to the tunnel, pores or voids can be seen on the advancing side of the SZ; this is due to excessive heat input [27]. The defects in the FS welds are sensitive to heat input which in turn depends on the parameters (tool rotational speed and traverse speed). Thus, the conditions of too high or too low heat input may result in defect formation. The macro-structure of Exp. No. 3 (Figure 3c) shows the presence of much smaller micro-cracks and crevices in the AS, which may be due to lower heat input than Exp. No. 1. No defect is visible for Exp. No. 4 (Figure 3d). The macrostructure shows a uniform and efficient flow of material due to the abundant stirring at high rotational speed and traverse speed that results in sufficient material mixing and optimal heat for material consolidation.

### 3.2. Microstructure

In order to analyze the microstructure of various FS welded samples, metallographic examinations were carried out. The visual examination showed that each specimen had undergone extensive grain refinement, especially in the nugget zone. The material beneath the shoulder softened due to the frictional heat between the tool shoulder and the top surface. Tool shoulder and pin/probe stirred the softened material. The state of the stirred material depended on the flow stress under prevailing temperature on both sides of the weld. There was a temperature and strain gradient from the AS to the retreating side (RS) of the weld seam, which lead to disparity in grain size distribution. 

**Figure 4:**
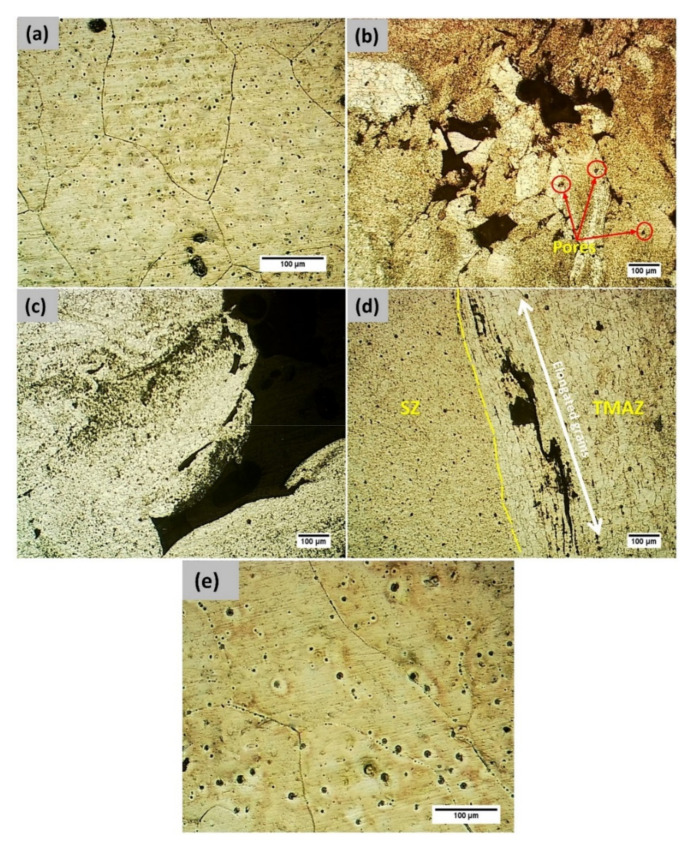
Microstructure corresponding to: (**a**) Base metal, (**b**) Tunnelling Defects and porosity corresponding to Exp. No. 1, (**c**) Tunnelling defect corresponding to Exp. No. 2, (**d**) Micro-cracks exhibited by Exp. No. 3, and (**e**) HAZ corresponding to Exp. No. 4.

**Figure 5:**
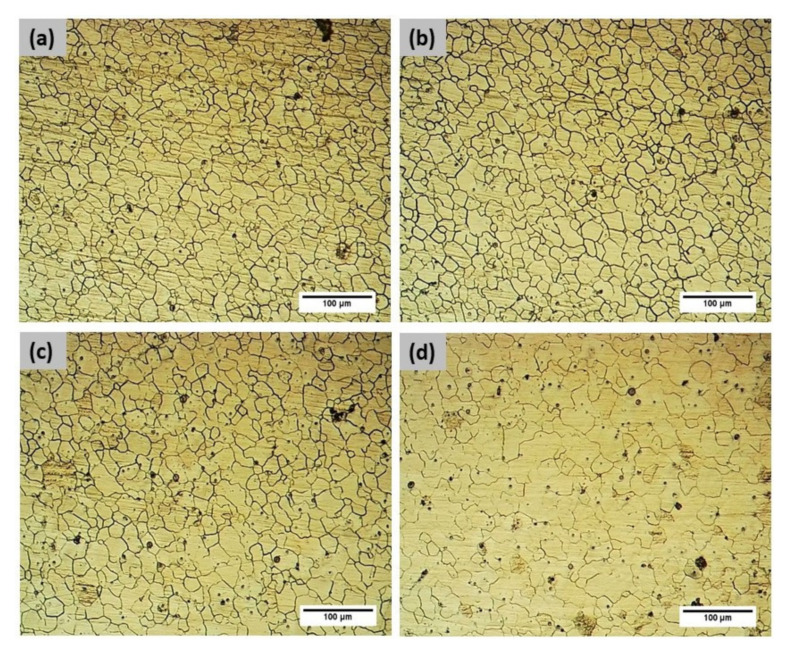
(**a**) SZ-AS for Exp. No. 4, (**b**) SZ-Centre for Exp. No. 4, (**c**) SZ-RS for Exp. No. 4, and (**d**) TMAZ for Exp. No. 4.

Figure 4a captures the microstructure of the base alloy, while Figure 4b–d show the presence of defects in welds corresponding to Exp. Nos. 1, 2, and 3, respectively. Exp. Nos. 1 and 2 show the presence of a tunnel defect on the advancing side; in addition to that, Exp. No. 1 also exhibits pores on the same side which is also evident from the macro-structure. The softened material in front of the tool during stirring is conveyed through 180° along the longitudinal direction in order to settle on the back of the tool [28]. Consequently, the material in front of the tool in the AS is subjected to greater displacement in order to be deposited in the AS behind the tool. Such transport mechanism leads to material deficiency in the AS of the weld seam, which can be controlled by the process parameters. In each of the welded samples, the prime contributor for the formation of defects is either excessive or insufficient heat input [29]. In comparison to Exp. No. 2, Exp. No. 3 is performed with lower traverse speed and the same rotational speed and consequently offers higher frictional heat input, which improves the weld surface. This leads to the formation of only the micro-cracks and crevices, as opposed to larger tunnels. Figure 5a–d correspond to SZ-AS, SZ-Centre, SZ-RS, and TMAZ of Exp. No. 4, clearly demarcating the grain refinement and grain size variation in the weldment. The average grain size in the SZ is found to be 16 ± 2 µm, while the average grain size of the base material was measured as 242 ± 15 µm.

### 3.3. Micro-Hardness

**Figure 6:**
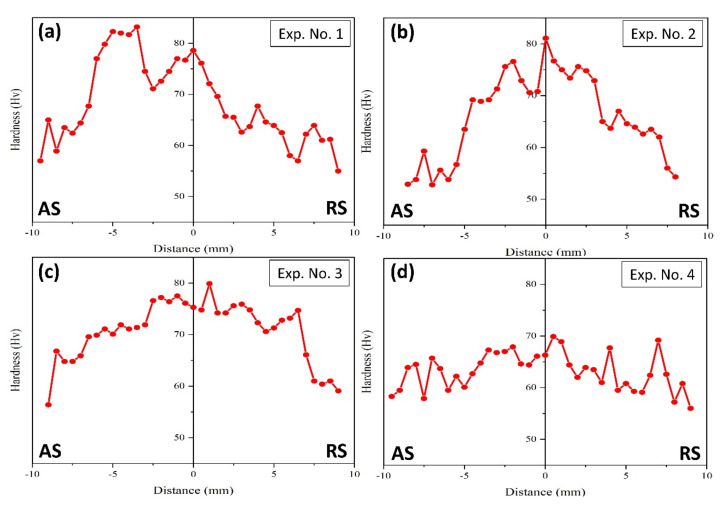
Micro-hardness distribution plots across the transverse cross-section of the welds for (**a**) Exp. No. 1, (**b**) Exp. No. 2, (**c**) Exp. No. 3, and (**d**) Exp. No. 4.

The micro-hardness distribution corresponding to Exp. Nos. 1–4 is shown in Figure 6. All welded samples showed higher micro-hardness values in SZ and TMAZ, which is attributed to a smaller grain size than the parent material. The micro-hardness values are subjected to a measurement uncertainty of ±1.5 HV in the SZ and ±3.0 HV outside the SZ. The material around the tool pin and near the shoulder experiences a higher degree of stirring, resulting in a greater degree of grain refinement in the SZ through severe plastic deformation and dynamic recrystallization. All the welds, except for Exp. No. 4, showed a similar distribution of the micro-hardness values, i.e., a decreasing pattern from SZ to HAZ on either side of the abutting line. This difference in hardness is due to the variation in grain size found in different zones. Unlike the ultra-refined and equiaxed grains in SZ, grains in TMAZ are elongated, and those in HAZ are coarser than the parent material. Unlike SZ, the grains in HAZ are subjected to thermal cycles, which promote grain growth.

**Figure 7:**
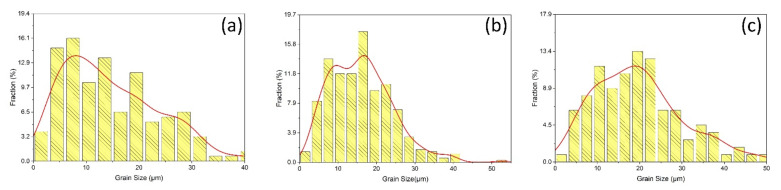
Grain size distribution for Exp. No. 4 (**a**) SZ-AS, (**b**) SZ-Centre, and (**c**) SZ-RS.

Further, the micro-hardness results for Exp. No. 1 shows comparatively higher values in the AS of the weld bead. Plastic deformation in a hot state promotes dynamic recrystallization and grain refinement [30]. The Exp. No. 1 is performed at high tool rotational speed which results in more DRX due to higher SPD and high heat input. Interestingly, the micro-hardness distribution corresponding to Exp. No. 4 shows fluctuation in on both sides of the abutting line. This fluctuation can be related to the grain size distribution in the SZ. Figure 7 demonstrates the grain size distribution for SZ-AS (Figure 5a), SZ-Centre (Figure 5b), and SZ-RS (Figure 5c) corresponding to Exp. No. 4. The local heterogeneity or variation in grain size and grain modality is evident through Figure 7a–c, being the prime reason for the fluctuation in micro-hardness. Such heterogeneity results from disparity in material transport mechanisms which are governed by tool rotational and traverse speeds for a fixed tool profile. In addition, the cam tri-flute tool with threaded profile generally promotes such heterogeneity.

### 3.4. Tensile Strength

**Figure 8:**
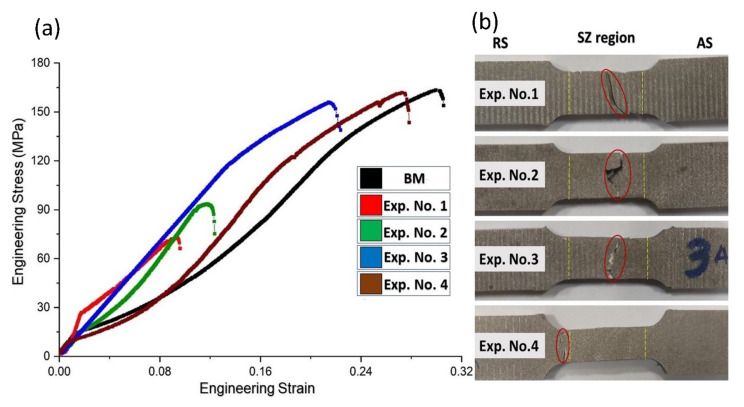
(**a**) Engineering stress vs Strain curves for Exp. Nos. 1–4 (**b**) Failed tensile specimen for Exp. Nos. 1–4.

To study the influence of the process parameters on strength of joints, Taguchi’s L4 orthogonal array was employed. The engineering stress–strain curves from the tensile tests of the base material and transverse specimens machined from the FS welded plates obtained at different parameters are shown in Figure 8. On observing the tensile test results of all the FS welds (Figure 8a), a significant disparity in the tensile strength of the individual welds is evident. As depicted from Figure 8a, the Ultimate Tensile Strength (UTS) and elongation at the maximum load for the base material are 163.5 MPa and 30.56%, respectively. The joint efficiency (i.e., the ratio UTS of welded joint and the base material) for Exp. No. 4 is 98.7%, and the elongation obtained is 91.07% of the base material.

In general, the FS joints are subjected to tensile failure at the TMAZ, HAZ, or at the interface of TMAZ/SZ of the advancing side of the joint [31]. However, the position of the fracture of the joint additionally relies upon the process parameters. As reported by Commin et al. [30], the fracture chiefly occurs at the transition of SZ/TMAZ on AS but may also occur on RS of the joint depending upon the relative values of tool rotational and traverse speed.

The welds corresponding to Exp. Nos. 1 and 2 exhibited low tensile strength and failed at the stir zone on the advancing side due to the presence of defects. However, weld from Exp. No. 3 also failed from the same location, and it showed a much higher tensile strength since the detrimental effect of microcracks is much less than that of the large tunnels. The defects induced in the welded samples result from either low or excessive heat input. A high rotational speed and low traverse speed results in excessive softening of the material, which promotes the flow of material outside the high-pressure weld zone, creating a material deficiency. In contrast, low rotational speed and high traverse speed lead to inadequate heat, adversely affecting interface bonding between the abutting plates. Thus, both conditions lead to a decrease in tensile strength [31]. Results indicate that the failure for Exp. No. 1 propagated along the pores and tunnel. The specimen obtained in Exp. No. 4 failed from the HAZ/Parent material transition on the RS due to larger grain size of HAZ than the other zones. As the respective macro-structure shows efficient material mixing and no defect in the specimen, the tensile test results of the specimen are found in its agreement.

## 4. Conclusions

This work investigates the feasibility of FS welding for joining 11.5 mm thick magnesium alloy plates, with an ultimate tensile strength of 163.5 MPa. Pertinently, single-pass FSW was employed in joining the magnesium plates effectively. The microstructural and mechanical properties of the welded joint were investigated, and the following conclusions can be drawn: 

Single-pass FSW can successfully join thick magnesium plates of thickness 11.5 mm, with no defects and sound weld integrity.

1. The optimization of process parameters can eliminate the chances of tunneling defect formation due to improved material flow and consolidation.
2. The grain refinement achieved in SZ is nearly 15 times the parent material, this is attributed to the degree of stirring and the tool profile used. An increase of 14.3% was achieved in the value of micro-hardness. Disparity in grain size distribution and grain modality across the stir zone leads to fluctuation in micro-hardness for the defect free weldment.
3. A joint efficiency of 98.9% and elongation of 27.87% was achieved at a rotational speed of 710 rpm and traverse speed of 100 mm/min. The fracture occurred at the interface of HAZ and parent material for this weld, ascertaining the perfect weld formation in the nugget zone.
4. Micro-crevices were observed at the SZ/TMAZ interface for tool rotational and traverse speeds of 560 rpm and 80 mm, respectively. In comparison to the tunneling defects, formation of these micro-crevices is far less detrimental to the strength of fabricated friction stir welds.

## Figures and Tables

**Table 1:** Chemical composition of the major elements and mechanical properties of the Mg alloy.

Elements (wt. %)	Mg	Y	Gd
	93%	4%	3%
Mechanical Properties	Tensile Strength (MPa)	Micro-hardness (HV)	Elongation (%)
	163.5	60	30.56

**Table 2:** Experimental Design Utilized for FSW.

Experiment No.	1	2	3	4
Rotational Speed “*ω*” (rpm)	710	560	560	710
Traverse Speed “*V*” (mm/min)	80	100	80	100

## Data Availability

All the data is available in the manuscript.
